# Tannins of Conifer Bark as Nordic Piquancy—Sustainable Preservative and Aroma?

**DOI:** 10.3390/molecules25030567

**Published:** 2020-01-28

**Authors:** Jan-Erik Raitanen, Eila Järvenpää, Risto Korpinen, Sari Mäkinen, Jarkko Hellström, Petri Kilpeläinen, Jaana Liimatainen, Ari Ora, Tuomo Tupasela, Tuula Jyske

**Affiliations:** 1 Natural Resources Institute Finland (Luke), Tietotie 2, FI-02150 Espoo, Finland; jan-erik.raitanen@helsinki.fi (J.-E.R.); risto.korpinen@luke.fi (R.K.); petri.kilpelainen@luke.fi (P.K.); jaana.liimatainen@luke.fi (J.L.); ari.ora@luke.fi (A.O.); 2 Department of Chemistry, University of Helsinki, PO Box 55, FI-00014 Helsinki, Finland; 3 Natural Resources Institute Finland (Luke), Myllytie 1, FI-31600 Jokioinen, Finland; eila.jarvenpaa@luke.fi (E.J.); sari.makinen@luke.fi (S.M.); jarkko.hellstrom@luke.fi (J.H.); tuomo.tupasela@luke.fi (T.T.)

**Keywords:** aroma, antioxidative, bark side-stream, flavor, phenolic compounds, preservative use, condensed tannins

## Abstract

Bark of Norway spruce and Scots pine trees contain large amounts of condensed tannins. Tannins extracted with hot water could be used in different applications as they possess antioxidative and antimicrobial activities. The use of bark tannins as e.g., food preservatives calls for increases in our knowledge of their antioxidative activities when applied in foodstuffs. To assess the ability of bark tannins to prevent lipid oxidation, hot water extracts were evaluated in a liposome model. Isolated tannins were also applied in dry-cured, salty meat snacks either as liquid extracts or in dry-powder form. Consumer acceptance of the snacks was tested by a sensory evaluation panel where outlook, odor, taste, and structure of the snacks were evaluated and compared to a commercial product without tannin ingredients. Our results show that conifer bark tannin-rich extracts have high capacity to prevent lipid oxidation in the liposome model. The efficacies of pine and spruce bark extracts were ten to hundred folds higher, respectively, than those of phenolic berry extracts. The bark extracts did not significantly influence the odor or taste of the meat snacks. The findings indicate that bark extracts may be used as sustainable food ingredients. However, more research is needed to verify their safety.

## 1. Introduction

**Figure 1:**
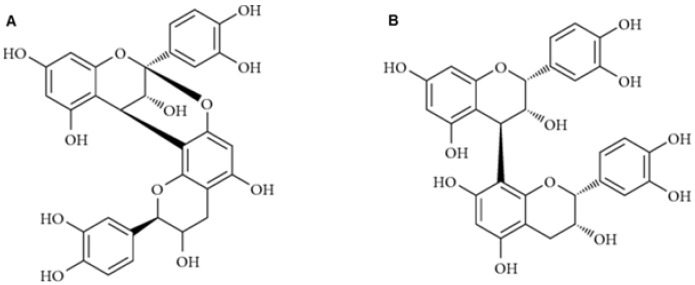
Structures of dimeric proanthocyanidins with A- and B-type linkages.

Tannins are ubiquitous polyphenolic compounds found in several plant species. Tannins form colorful pigments, and they cause astringent and bitter taste for fruits, plants, and bark. Bark of coniferous Norway spruce (*Picea abies* [L.] Karst.) and Scots pine (*Pinus sylvestris* L.) trees contains high amounts of condensed tannins (CTs), also called proanthocyanidins (PAs) [1]. Condensed tannins are oligomers or polymers of flavan-3-ol units linked by carbon-carbon bonds that resist hydrolysis [1,2]. The most common PAs are procyanidins (PCs) and prodelphinidins (PDs). PCs consist of catechin and/or epicatechin units. PDs consist of (epi)gallocatechin units [1,2]. In Norway spruce bark, both PCs and PDs exist [3]. In contrast, in Scots pine bark only PCs are observed [3]. Flavan-3-ol units are typically linked by B-type bonding (C_4_ → C_8_ or C_4_ → C_6_ linkages), while A-type bonds occur more rarely (additional C_2_ → O_7_ or C_2_ → O_5_ linkages) [4] (Figure 1).

In vascular plants, phenolic tannins function as defensive secondary compounds. Tannins provide protection against UV damage caused by sunlight, or oxidative side reactions in the cellular milieu, preventing harmful radicals from destroying the cellular structures [5,6,7]. In coniferous bark, tannins protect trees from being infected by bacteria and fungi.

Tannins express antioxidant activity through several mechanisms, i.e., free radical scavenging activity, the chelation of transition metals, and inhibition of prooxidative enzymes [8]. Tannins have a well-known ability to bind proteins and other compounds, and form complexes with them, this propensity believed to be a reason behind their interactivity with biological systems [4,9,10]. Tannins also act as antimicrobial agents, expressing antimicrobial activity via different mechanisms, such as inhibition of extracellular microbial enzymes, deprivation of the substrates required for microbial growth, and direct action on microbial metabolism e.g., denaturation of proteins of cell membranes [11]. Due to these biological activities, a growing commercial interest towards tannins exists [12]. Tannins could be used, e.g., in adhesives, functional coatings, or as preservatives in different targeted applications, such as against food spoilage, and even as flavor compounds in food [13,14].

Conifer bark is a major industrial byproduct in the Nordic countries, being one of the most prominent resources for added-value biochemical production in boreal biocircular economy. In Finland only, the forest industry uses ca. 70 million m^3^ of round wood annually [15]. As the amount of bark is approximately 10% of the round wood volume, the industry produces ca. 7 million m^3^ of bark as a byproduct. This residue is mainly combusted for energy production. Transformation into biocircular economy requires for more efficient and comprehensive utilization of bark [16]. For higher added value purposes, bark PAs can be extracted by using environmentally benign solvents, such as pure hot water [3].

Lipid oxidation in meat is a significant problem causing meat food deterioration [17]. It is related to the formation of off-flavors and off-odors reducing the food quality, due to the autooxidation mechanism and formation of free radicals, which are usually prevented by using additives in manufacturing processes of meat products, e.g., sausages. For cured sausages, only table salt together with nitrates and nitrites are accepted additives in the EU, however, added spices and flavors may interfere with the fat oxidation processes. The use of nitrites and other synthetic antioxidants is discouraged in the industry due to their potential harmful effects especially on the health of young children, and natural antioxidants are actively searched as substitutes [18,19,20].

This study explored the potential of spruce and pine bark-derived PA-rich extracts for developing preservative agents for the food industry, to provide protection from lipid oxidation of e.g., fatty-acids containing meat products. For this purpose, PAs were extracted from Norway spruce and Scots pine bark, and the lipid peroxidation inhibition capacity of the extracts was studied by using a liposome model. PAs could also serve as a flavoring agent in foodstuffs and/or affect the sensory properties of the food. The customer acceptance of the tannic taste of bark-obtained tannins is not known. Thus, the extract that showed high lipid oxidation inhibition capacity in our tests (i.e., Norway spruce whole bark extract) was also applied into fermented dry- and salt-cured reindeer meat snacks. The consumer acceptance of the meat snacks with tannin addition was then studied by sensory evaluation in which flavor, odor, and pleasantness of the meat snacks were tested.

## 2. Results and Discussion

### 2.1. Composition of Bark Raw Materials

**Figure 2:**
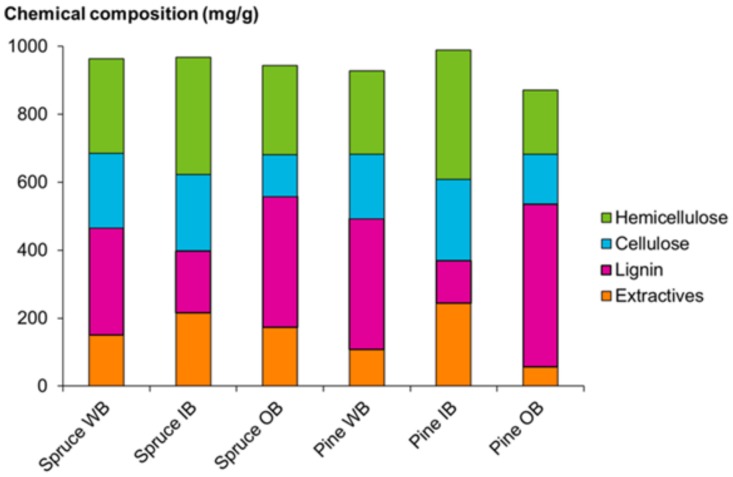
Composition of bark raw materials. WB, whole bark; IB, inner bark; OB, outer bark.

The chemical composition of the bark raw materials of spruce and pine is shown in Figure 2. Lignin concentration was 31% for spruce whole bark (WB) (dw) and 38% for pine WB, while the outer bark (OB) fractions showed higher lignin content than inner bark (IB). In contrast, cellulose and hemicellulose concentrations were higher for IB than for OB in both species, while the WB contents of cellulose and hemicellulose were 22% and 28% for spruce, and 19% and 25% for pine, respectively.

The total concentration of bark extractives was 15% for spruce WB and 11% for pine WB. In IB and OB, the corresponding values were 22% and 17% for spruce, and 24% and 6% for pine, respectively. The majority of the extractives detected in both tree species were hydrophilic compounds, i.e., 59–84% of the total amount of extractives, the value depending on the bark fraction.

### 2.2. Yield and Composition of Bark Extracts

#### 2.2.1. Extraction Yield

**Figure 3:**
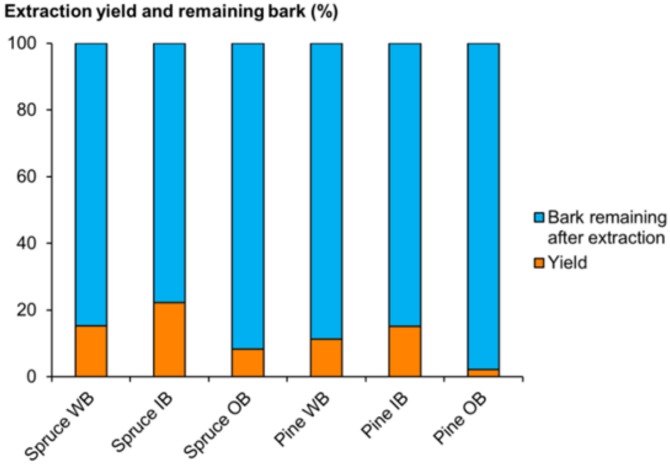
Extraction yield and bark remaining after extractions. WB, whole bark; IB, inner bark; OB, outer bark.

The yields of total dissolved solids (TDS) in hot water extractions of whole bark (WB) were 15.2% (*w*/*w*) for spruce and 11.2% (*w*/*w*) for pine (Figure 3). The yields of TDS for inner bark (IB) and outer (OB) were 22.2% and 8.2% for spruce; and 15.1% and 2.2% for pine, respectively (Figure 3). Our results are in accordance with earlier findings. Our previous study reported that the yield of TDS in the hot water flow-through extraction of oven dry bark was 8.1–8.8% (*w*/*w*) for Norway spruce and 6.2–6.5% (*w*/*w*) for Scots pine [16].

Hot water extracted 81.2 mg/g and 36.0 mg/g of tannins from spruce WB and pine WB (dw), respectively. For both tree species, tannin yields were higher for inner bark (IB) than for outer bark (OB) (spruce IB 91.9 mg/g vs. spruce OB 48.2 mg/g; pine IB 41.2 mg/g vs. pine OB 10.6 mg/g). This study did not focus on the optimization of tannin yields in extraction and purification, which remains as a topic for further studies to feasibly produce high-value tannin-based ingredients for targeted applications. Our results are in accordance with previously published research. According to a previous study [3], the content of water-soluble PCs and PDs in spruce bark was 3.6% and 0.08% (dw), respectively. The same authors reported pine bark to contain 3.1% and 1.0% (dw) of non-water-soluble PAs and water-soluble PCs, respectively. In their study, the water-soluble compounds mainly originated from the inner bark, similarly to our findings. In another study, industrial, wood-free spruce bark contained 10.7% (*w*/*w*) of tannins [21]. The authors found industrial bark to contain a high amount of wood (ca. 20%), and bark-wood mixture showed a slightly lower tannin content (8.3%, *w*/*w*) than wood-free bark.

#### 2.2.2. Chemical Composition of Bark Extracts

**Figure 4:**
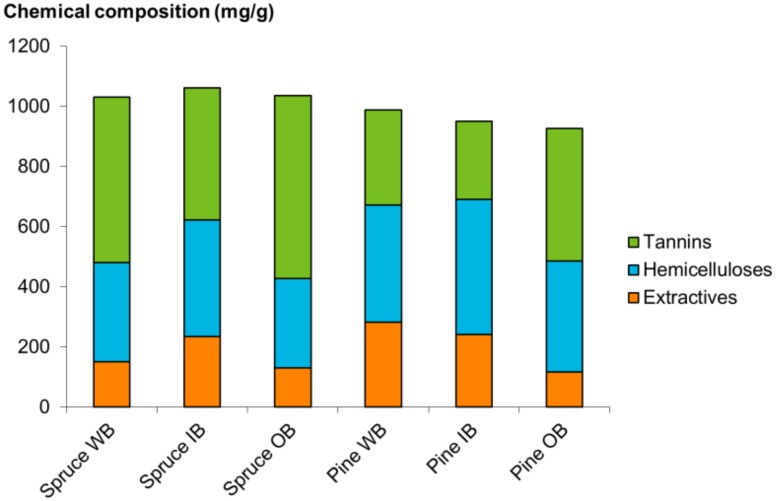
Chemical composition of bark extracts. WB, whole bark; IB, inner bark; OB, outer bark.

The composition of hot water extracts of spruce and pine is shown in Figure 4. For both spruce and pine, the proportion of tannins in the extracts was the highest in the outer bark (59% for spruce and 48% for pine), and somewhat lower in inner bark (41% for spruce and 27% for pine). For whole bark, the tannin proportion of the total extract composition was 53% and 32% for spruce and pine, respectively. In “pettu”, tannins comprised of 29% while hemicelluloses were major compounds (data not shown). In bark extracts, the content of other extractives varied from 15% in spruce WB to 29% in pine WB. The spruce compounds consisted of sugar alcohols, organic acids, phenolic compounds/lignin fragments, resin acids/alcohols, stilbene glucosides, and unidentified compounds. In pine bark extracts, the composition was similar excluding the resin acids and stilbene glucosides. In “pettu”, the content of extractives other than tannin and hemicelluloses was not quantified because “pettu” did not dissolve in water.

#### 2.2.3. Chemical Composition and Properties of Condensed Tannins

PAs in Norway spruce bark extracts were mostly composed of (epi)catechins but also (epi)gallocatechins were detected as minor subunits (Table 1). PAs in Scots pine bark extracts were essentially (epi)catechins, i.e., PCs. The results are in agreement with previous studies [3,22,23]. Scots pine bark was reported to contain up to 5 g/100 g (fw) of extractable PAs [3,4]. According to Matthews et al. [3], the mean degree of polymerization (DP) of pine bark PCs was 5.3. PAs in Norway spruce bark were mainly PCs (98%) with only 2% of PDs, and the mean DP was 4.6 [3]. Bianchi et al. [24] reported similar results for bark PAs with slightly higher DP for pine bark and spruce bark: 6.7 and 6.2, respectively. In our study, the mean DP in spruce bark was 4.8, and that of pine bark 3.5.

Whole bark extracts of Scots pine had 2.7-times higher CT content than that of Norway spruce bark (*p* > 0.001; Table 1). In Norway spruce, the inner bark extract had 45% higher (*p* > 0.001) CT content than the outer bark extract but in Scots pine, no differences were found between the inner and outer bark extracts. The commercial inner bark powder of pine, “pettu”, showed 14% higher CT content as compared to the inner bark -derived extract (Table 1). These results are contradictory to the results of Folin-Ciocalteu method showed in the Section 2.2.2; however, that method is more robust as compared to the analysis of CTs by HPLC after thiolytic degradation.

Krogell et al. [25] extracted tannins from Norway spruce bark with hot water and the CT contents in the extracts were very similar with the contents determined in the present study. 24. Bianchi et al. [24] compared hot water extracts of barks of different softwood species and the reported tannin content in Scots pine bark extract is well in accordance with our results. However, they stated clearly higher tannin content for Norway spruce bark extract than we found in the present study. This is most likely due to different analytical methods. In the present study, PAs were determined by thiolysis and only proanthocyanidins, i.e., flavan-3-ol polymers, were included in the PAs while Bianchi et al. [24] used total phenol assay for quantification after fractionating phenolics to monomers and polymers. They reported that only 26% of polymers in Norway spruce bark extract were quantifiable by thiolysis, indicating that majority of the polymer fraction was actually not composed of proanthocyanidins. The results achieved by thiolysis agree well with each other in both studies.

### 2.3. Inhibition of Lipid Oxidation by Tannin Extracts in a Liposome Model

**Figure 5:**
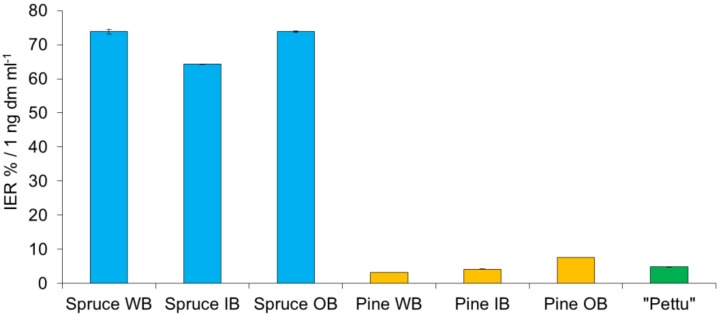
Lipid oxidation prevented by the Norway spruce and Scots pine tannin-rich bark extracts as analyzed by a liposome model. WB, whole bark; IB, inner bark; OB, outer bark; Pettu, commercial inner bark powder of pine.

The inhibition of lipid oxidation by PA-rich extracts, as analyzed by a liposome model, was high: on average 70.6 IER %/1 ng dm/mL for spruce, and 5.1 IER %/1 ng dm/mL for pine. The spruce extracts had on average 14-times higher inhibition capacity as compared to that of pine extracts (*p* < 0.001; Figure 5). Outer bark extracts of spruce and pine showed significantly (15% and 83%, respectively, *p* < 0.001) higher inhibition capacity in comparison to inner bark extracts. The commercial “pettu” showed 15% higher inhibition capacity than pine inner bark-derived extract (Figure 5).

The significantly higher inhibition capacity of spruce in comparison to pine may be due to different chemical composition of the extracts. Spruce extracts contained 3% (WB), 9% (IB), and 2% (OB) of stilbene glucosides, and 2% (WB) and 1% (OB) of resin acids, which were absent in the pine extracts. These results are in accordance with the previously reported ones [14,25,26,27,28,29].

Glycosylated monomeric stilbene glucosides (astringin, isorhapontin, and piceid) are structurally similar to by far the most extensively studied stilbenoid, *trans*-resveratrol, for which there is a vast amount of accumulated scientific evidence on its multiple biological activities, including antioxidant [30]. The bark extract of Norway spruce has been shown to exhibit strong antioxidant activity against lipid peroxidation [31]. The extract has also been shown to possess antimicrobial activities against diverse pathogenic, food, and agricultural microbes [32]. *P. mariana* (black spruce) bark extract and two of its PC fractions have also been shown to exhibit anti-inflammatory and free radical-scavenging activity *in vitro* [33].

Lipid oxidation inhibition capacities reported in the literature concern mainly berry phenolics. For example, lipid oxidation inhibition of 25–51% at a sample concentration of 1.4 µg dm/mL has been reported for the phenolic extract of raspberry, lingonberry, and bilberry [34]. In comparison to the berry phenolic extracts, the efficacies of the pine and spruce bark samples of the present study were ten to hundred folds higher, respectively. The outer bark samples prevented lipid oxidation slightly more effectively than the inner bark samples; however, the difference was not statistically significant.

Vuorela et al. [35] suggested that pine bark (*P. sylvestris*) could be a potential source of antioxidants for meat products. The authors studied oxidation of cooked pork meat with an added bark extract for 9 days at 5 °C under light. The bark extract was added at a level preventing lipid oxidation by > 80%, this was 10.6 mL (containing 8.1 mg of phenolic compounds) / 100 g of meat. The oxidation was followed by measuring the formation of hexanal and the formation of protein-derived carbonyl compounds. Hexanal formation inhibition was 98.2% and protein carbonyls 63.5%. They concluded that pine bark constituent taxifolin was an effective antioxidant against protein oxidation, but was not the only compound responsible for the antioxidant activity of pine bark as several other compounds, such as lignans and catechins, were present [35]. Vuorela et al. [36] showed that Scots pine bark fractions were antioxidants against the oxidation of liposomes and LDL particles. The same authors concluded that phenolic isolates from pine bark are safe and bioactive for possible food applications including functional foods intended for health benefits.

Iglesias et al. [37] compared the ability of PC fractions of pine bark extract (*P. pinaster*) and grape pomace to inhibit lipid oxidation in fish lipid systems. The homologous pine bark and grape pomace PC fractions had similar polymerization degrees, but differed in the galloylation, since esterified galloyl groups were absent in pine PCs. The addition of 100 µg/g of PC fraction to bulk fish oil showed that the lowest polymerized fractions were the most efficient antioxidants, while the galloylation did not have influence on the activity. In fish oil-in-water emulsions, the intermediate DP (mDP = 2.2) showed the highest antioxidant activity and galloylated fractions were more effective in inhibiting oxidation [37]. Touriño et al. [38] showed the same general trend that galloylated PC fractions were the most effective against lipid peroxidation in corn oil emulsion. However, they noted that galloylation had little influence on the capacity of oligomeric PCs (2 ≤ DP ≤ 4) to protect lipids from peroxidation [38].

Scots pine bark extract has been shown to have high antioxidant activity in the methyl linoleate model [39], high antimicrobial activity against *Staphylococcus aureus* [40], and it contains compounds that inhibit the production of two proinflammatory mediators, nitric oxide and prostaglandin E2 [36,41]. Scots pine phloem aqueous methanol extract has been shown to be antimicrobial against *S. aureus*, one of the most common bacteria causing food poisoning [40]. Further, the extract showed slight inhibiting activity against *Escherichia coli* and the yeast *Candida albicans* [40].

### 2.4. The Treatment of Meat Snacks

For the preparation of reindeer meat snacks with tannin addition, CT-rich whole spruce bark extract was chosen due to its high lipid inhibition capacity and the most feasible pretreatment requirements for hot water extraction (i.e., no need to separate inner and outer bark before grinding). After pre-trials with 0.1% and 1% tannin extract additions, preliminary sensory analysis was carried out. Based on the results, the product with 1% extract was preferred among men but the snack with 0.1% got higher scores among women. Thus, the large scale preparation of meat snacks for analysis was done by using a 0.5% concentration of dried extract that was mixed together with the ingredients before fermentation. Additionally, after fermentation, the extract was prepared into an aqueous solution (with an estimated tannin concentration in final product varying between ca. 0.2–0.4%) and sprayed on the dry-cured, sliced snacks without other tannin addition.

### 2.5. TBA of the Meat Snacks

The TBA-value for the meat snacks with tannin addition was on average 2.23 mg TBA / kg product (mean for the two identical products extract 1 and extract 2), being similar to the value of control product (2.28 mg TBA / kg product). The TBA-value for the meat snack with sprayed tannin extract (3.17 mg TBA / kg product) was 39% higher than that of control. This may be due to more uniform structure and spread of the sprayed extract on the snacks. Other studies have shown that tannic acid acts as an effective natural preservative (lipid and protein oxidation, color, and volatiles were analyzed) in cooked chicken meat, thus preserving the quality during storage [18]. Similarly, plant-derived extracts and materials were shown to provide protection against lipid oxidation in deep-fried meatballs and by using a meat model system [19,20]. The highest lipid oxidation inhibition capacity in meatballs was found for summer savory (*Satureja hortensis* L.) lyophilized powder and sea buckthorn leaf extract: the lipid oxidation was reduced to 14% and 23% at the 100 mg/kg concentration, expressed as gallic acid equivalents, respectively [19]. In the meat model tests, summer savory lyophilized powder also showed the highest inhibition capacity (oxidation was reduced to 17% compared to control), while spruce inner bark extract obtained from a twostep extraction using hexane and 95% ethanol (aq) was also tested, and oxidation of the samples was reduced to 19% compared to control, at 200 ppm after 2 weeks [20].

### 2.6. Sensory Assessment of Meat Snacks with Tannin Additions

Sensorial characteristics play a pivotal role in food acceptance, preferences and choices. Tannin rich foods are essentially characterized by two major sensorial aspects: bitterness and especially astringency [42]. Astringency is a tactile sensation that is associated with the ability of certain chemicals to interact/bind and precipitate salivary mucoproteins that normally lubricate the mouth. In our study, the meat snacks that were modified with tannin extracts of spruce bark appeared to have a new flavor/aroma as compared to the control snacks without tannin (Table 2). On average, the snacks with extract 2 (i.e., added in dry form before fermentation) were found to be tastier and have more pleasant smell than the other snacks with or without extract. However, the differences in taste and smell between the tested snack types were statistically not significant (*p* > 0.99). In general, all the products achieved very similar scores for all the tested properties as the gender-wise averages for the property scores varied from 2.92 to 4.12 (SDs for different properties varied between 0.35–1.20).

Different profiles for outlook, smell, taste, and structure were obtained by adding the extracts to the meat before/after fermentation. On average, maximum taste and smell scores were achieved when tannin was added before fermentation by using a dry-type extract (i.e., extract 2). The best scores for outlook and structure; on the other hand, were given for snacks named as extract 1 (i.e., the same product as that with name extract 2; tannins added in dry-powder form before fermentation) and the sprayed extract on the fermented product, respectively.

In general, women liked the smell of all the products more than men (*p* < 0.01). For the taste and smell, the results between the snack types were consistent between both genders of the sensory panel members (i.e., best scores for snacks named as extract 2). However, non-statistical gender-trends were found regarding the best scores for the outlook and structure of the snacks: women preferred the products named as extract 1, while men gave the best scores for the snacks without tannins (i.e., control) and with the sprayed extract, respectively (Table 2).

The willingness to buy the products was at much higher level among the male than among the female panelists (Table 2). On average, 67% and 75% of the males were willing to buy the snacks with and without (i.e., commercial control) tannin addition, respectively. From the females, only 17% and 38% were interested in buying the same products. The results may be related to the cultural differences between genders in meat products consumption in Finland. Meat products are currently more typically consumed/a more common part of the diet among males as compared to the female population in Finland [43].

In our study, the preliminary tests with meat product prototypes consisted of tannin extracts with concentrations of 0.1% and 1% in the products by using both dried and liquid type extracts. The male panelists did not seem to find the level of astringency and bitterness too high as they preferred the taste of higher concentration over the lower extract concentration. For women in contrast, the preferred extract concentration was the lower one. Several studies have examined the relationship between wine or grape extract polyphenol/PAs sensory properties and their structure [44,45,46,47]. Whereas it is clear that the perception of astringency increases with tannin concentration on a mass basis and with increasing molecular size, i.e., DP, detailed and convergent information about particular sub attributes is still lacking [48]. Peleg et al. [49] compared the bitterness and astringency of aqueous solutions of (+)-catechin and (‒)-epicatechin monomers and their five synthetic oligomers (three dimers and two trimers). They noted, as well, that as the molecular size increased the bitterness decreased and astringency increased [49].

### 2.7. Safety of Bark-Derived Flava-3-ols and Proanthocyanidins in Foodstuffs

Proanthocyanidins are found in many common foodstuffs and they have a long history in human diet [50]. Flavan-3-ols and especially epigallocatechin gallate are common in green tea and green tea preparations. Green tea flavan-3-ols have been implicated to have both beneficial and harmful effects on human health [51]. Few toxicological studies have been made with the purified individual flavan-3-ols. Studies have demonstrated the lack of mutagenic activity of several flavan-3-ols in bacterial reverse mutation tests [52,53,54,55]. Epigallocatechin was shown to exhibit weak mutagenic activity [52,55]. Several pure procyanidins including dimers, a trimer and a polymer fraction were shown to be non-mutagenic in the Ames test [56].

However, proanthocyanidin rich dietary supplements and fortified foods may lead to a multifold intake of these polyphenols compared to a normal diet [57]. Especially the use of coniferous bark-derived ingredients as preservatives and/or aroma compounds in food need more research on safety. Novel food authorization is required, unless the plant or plant part has been used as food to a significant degree prior to 1997 within the EU. The approval of novel foods, when introduced for the first time to the food market in the EU, requires an application procedure. The procedure includes clarifications on, e.g., safety and composition of the product, raw material, and production process, Hazard Analysis and Critical Control Points (HACCP), potential harmful substances, estimated dietary intake, frequency of use, and general safety. Harmful and allergenic molecules such as resin acids need to be taken into account. Regarding Norway spruce, only the use of the bud of young shoots (sprouts) is known as a food ingredient in Finland [58]. In EU, the use of the leaves (needles), flowers, cones, and resin of Norway spruce is known as food supplements. Based on the record of the Finnish Food Authority Ruokavirasto, “pettu” is not a novel food and may therefore be added to foods, at least in Finland; however, in the EU, pine sprouts and young needles, cones, and bark are allowed only in herbal tea or as food supplements [58].

Pine inner bark powder was used as flour extension in bread-making during the times of famine in Finland, such as severe frost and the famine years in the 1860 s [59]. Traditionally prepared “pettu” was cut from a trunk as a large bark cylinder and the outermost parts, brown and green periderm, were carved off. The harmful components were removed by roasting and scratching off the oozed substances, or by boiling the bark in water for 2–3 h. Finally, the bark was dried and ground. Traditionally, pine bark powder was mixed with flour of rye or other cereals up to 1/1 volume. The pure bark powder easily caused stomachache and constipation [59]. The bark flour was also mixed with milk, fat and blood of reindeer and fish/meat soup by Sami people [60]. In liquid foods, it has a thickening effect. Pine inner bark was not an emergency food for Sami people, but rather a valued staple food [60].

Pine phloem has high content of insoluble fiber and polyphenols such as lignans, catechins and procyanidins [61]. Consumption of phloem-fortified bread (70 g/day containing 62 mg catechins and procyanidin dimers) for four weeks was shown to increase the oxidation resistance of total serum lipids in humans [61]. Rats tolerated properly treated pine bark well when served as a traditionally common concentration (25% w/w, about 50% v/v): the observed smaller increase of body weight compared to control group corresponded to the lower energy content of the bark pellets [59].

Nowadays pine bark extracts are used as a nutritional supplement and traditional phytochemical remedy for various diseases throughout the world, including chronic inflammation, circulatory dysfunction, and asthma. These supplements are sold with trade names such as Pycnogenol, Oligopin (both extracted from *P. pinaster*) and Enzogenol (*P. radiata*). In addition, the bark of *P. massoniana* Lamb has been used in traditional Chinese medicine [62]. These pine bark extracts have PCs as their principal ingredients, but they also contain other pine bark phenolic constituents [63]. Pine bark extracts have been studied in several animal experiments and clinical trials and almost all reported them to be safe and well tolerated with a few side effects [63]. For example, Pycnogenol is reported causing gastrointestinal discomfort as the most frequently occurring adverse effect followed by dizziness, headache and nausea. The safety trials of Pycnogenol have demonstrated the absence of mutagenic and teratogenic effects, no perinatal toxicity, and no negative effects on fertility. Although there are no long-term safety studies, no serious adverse effects in any clinical study or from commercial use has been reported since it was initially introduced into the market in Europe around 1970 [64].

For Norway spruce bark, no literature was found on traditional oral usage. Furthermore, to the best of our knowledge, there is no toxicity data for Norway spruce bark extract. Acute oral toxicity of black spruce (*P. mariana*) bark hot water extract, instead, was tested on Sprague-Dawley rats in preliminary *in vivo* trials [65]. The black spruce extract showed no toxicity since its LD50 toxicity was greater than 2000 mg/kg. However, toxicity trials and safety assessments are species and extract specific. Thus, toxicity tests for Norway spruce bark extracts remain as a topic for further studies.

## 3. Materials and Methods

### 3.1. Chemicals

Unless otherwise stated, the chemicals were purchased from VWR International.

### 3.2. Bark Materials

Norway spruce (*Picea abies* [L.] Karst.) and Scots pine (*Pinus sylvestris* L.) bark samples were obtained from mature (36 and 63 years old, respectively) trees grown in Southern Finland, at Ruotsinkylä research forest of Natural Resources Institute Finland (60.2°N, 25.0°E, 60 m a.s.l.). Trees were harvested in early February and trunk poles transported into the laboratory and stored at −20 °C. Whole bark (WB), inner bark (IB) and outer bark (OB) were manually separated based on morphological and color differences between bark layers [66] and used in the experiments. For pre-trials, Norway spruce whole bark from Kuru research forest (61.5° N, 23.4° E) was used. The bark samples were ground using a cutting mill without a sieve cassette. The particle size of the milled bark was 2–15 mm. Commercial “pettu” was purchased from Hunajakioski (Heinäholman Mehiläistarhat, Nederlappfors, Finland). “Pettu” is a roasted powder obtained from the inner bark of Scots pine. It was used as flour extension in bread-making during famine in Finland [59].

### 3.3. Tannin Extraction

PAs were extracted from Norway spruce and Scots pine whole bark (WB), inner bark (IB) and outer bark (OB) using a 2-litre stirred reactor (Büchiglasuster, Uster, Switzerland). The extraction conditions can be seen in Table 3. Pure water was used as a solvent.

The tannin extract was discharged from the reactor through a cooling unit and bark was removed from the reactor and manually pressed inside a 50 µm filter bag (Eaton GAF, Eaton Technologies GmbH, Nettersheim, Germany). The liquids were combined and weighed. The total dissolved solids were determined from the extracts and they were freeze-dried to a fine powder using a freeze dryer (Christ Alpha 1-4 LSC, Osterode, Germany).

### 3.4. Chemical Composition of Bark

For the chemical analysis of bark composition, ground bark was first freeze-dried and then further milled with a Retsch MM400 ball mill (Retsch GmbH, Haan, Germany) into a smaller particle size of 0.5–1 mm for the analyses.

#### 3.4.1. Lipophilic and Hydrophilic Extractives

Lipophilic and hydrophilic extractives were removed using an accelerated solvent extraction (ASE-350) apparatus (Dionex, Sunnyvale, CA, USA). A stainless steel extraction cell (Dionex) was loaded with 6 g of raw material powder and extracted with *n*-hexane at 90 °C and the residue was again extracted with acetone/H_2_O (95:5, *v*/*v*) at 100 °C. The extractions were performed as 3 × 5 min static cycles. The final volume of the hexane and acetone/H_2_O-extract was adjusted to 50 mL. The extractives content was determined gravimetrically as well as by GC-FID or GC-MS analysis. Prior to all GC-analyses, aliquots of extracts were evaporated to dryness under N_2_-stream and silylated by adding 150 μL of a mixture of pyridine, N,O-bis(trimethylsilyl) trifluoroacetamide (BSTFA, Supelco Analytical, Bellefonte, PA, USA), and trimethylsilyl chloride (TMCS, Merck KGaA, Darmstadt, Germany) in a 1:4:1 (*v*/*v*/*v*) ratio, and the mixture was heated in an oven at 70 °C for 45 min. Betulinol (0.02 mg/mL) and heneicosanoic acid (C21:0, 0.02 mg/mL) served as internal standards. The silylated samples were analyzed on a GC-MS (HP6890-5973 GC-MSD instrument, Hewlett Packard, Palo Alto, CA, USA). The GC-column was an HP-5 column (Agilent Technologies, Inc., Santa Clara, CA, USA; 30 m × 0.25 mm i.d., film thickness 0.25 μm). The injector and MS interface temperatures were kept at 280 and 300 °C, respectively. Helium was used as carrier gas and the injection was made in splitless mode. Mass spectra were obtained in EI mode (70 eV) and the fragmentation pattern was compared to standards in commercial (NIST14/Wiley11) libraries, as well as the standards in our own MS libraries available at our laboratory.

#### 3.4.2. Hemicellulose Content

The hemicellulose content was determined by acid methanolysis-GC according to a previously reported procedure [67]. Sample of 8–12 mg of raw material, and 2 mL of a 2 M HCl solution (in anhydrous MeOH) was added in a pressure resistant pear-shaped flask (duplicate samples) and the sample was kept at 105 °C for 5 h. 1 mL of a calibration solution (duplicate samples) containing 0.1 mg/mL of each monosaccharide (arabinose, glucose, glucuronic acid, galactose, galacturonic acid, mannose, rhamnose and xylose) was evaporated to dryness and treated for 3 h in the same way as above. After cooling to room temperature, 200 μL pyridine was added to neutralize the acidic solution and 4 mL Resorcinol (0.1 mg/mL) was added as internal standard (IS). 1 mL of the clear phase was taken into a test tube and evaporated to dryness under N_2_-stream. The dry residue was silylated over night by adding 150 μL pyridine, 150 μL 1,1,1,3,3,3-hexamethyl disilazane (HMDS, Sigma-Aldrich Chemie GmbH, Steinheim, Germany) and 70 μL trimethylsilyl chloride (TMCS, Merck KGaA) and analysed by GC-FID (Shimadzu GC-2010, Kyoto, Japan) with HP-1 Column (25 m × 0.2 mm I.d., film thickness 0.11 µm). In the calculations of the results, a correction factor of 0.88 and 0.90 was used for pentoses and hexoses, respectively.

#### 3.4.3. Cellulose Content

The cellulose content was determined by acid hydrolysis-GC. A total of 10 mg of raw material (duplicate samples) was placed into a test tube together with a glass ball. 0.2 mL of H_2_SO_4_ (72%) was added and the test tube was briefly placed into a vacuum oven (Thermo Fisher Scientific, Waltham, MA, USA). The pressure was allowed to reach 0 mbar and go back to normal pressure. The test tube was then taken out and allowed to stand at r.t. for 2 h. 0.5 mL of H_2_O was added and the test tube was then allowed to stand at r.t. for 4 h. 6 mL of H_2_O was then added and the test tube was allowed to stand in a fume hood overnight. The following day the test tube was autoclaved at 120 °C for 1 h and then allowed to cool to r.t. BaCO_3_ was used to neutralize the solution and bromocresol green was added as indicator to monitor the change in colour from yellow to blue. 1 mL sorbitol (5 mg/mL in H_2_O) was added as IS and the test tube was centrifuged. An aliquot of the clear solution was transferred to a test tube and subsequently evaporated to dryness under N_2_-stream and silylated in the same way as the acid methanolysis samples. Cotton linters (duplicate samples) were used for calibration and treated in the same way as described above. The cellulose content was determined on the same GC-system as glucose. The cellulose content of the raw material was obtained by subtracting the value for glucose (anhydro) obtained from hemicellulose from the acid hydrolysis value.

#### 3.4.4. Lignin Content

The lignin content was determined as Klason lignin and acid-soluble lignin with the following pretreatment: the extractives-free bark residues (2 g) were first extracted with 3% KOH (*w*/*v*) in EtOH for 2 h at 70 °C to remove suberin and polymeric phenolic acids and then filtered. The residues were dried at 105 °C overnight. The total lignin content was determined on dry residues (0.5 g) as described by [68].

### 3.5. Chemical Composition of Tannin Extract Powders

#### 3.5.1. Carbohydrates

Carbohydrates in the extracts were analysed by acid methanolysis as described earlier. Monomeric sugars in the extracts were analysed by adding 1 mL internal standard containing 0.1 mg/mL xylitol in MeOH/water (9:1) into a sample containing 2 mg freeze-dried material. The solvent was evaporated under N_2_-stream and the dried sample was dissolved in 150 μL pyridine. The dissolved sample was derivatized with 150 μL HMDS and 70 μL TMCS and analyzed by GC as described earlier.

#### 3.5.2. Extractives

Exactly 250.0 mg tannin powders were weighed and transferred into 25 mL volumetric flasks. The flasks were filled with 25 mL water (10 mg/mL). The flasks were placed into an ultrasound bath for a short period in order to obtain a homogeneous solution. 50 µL of the solution was pipetted into a test tube. The content was then freeze-dried. 0.1 mL internal standard was added (0.5 mg/mL), and the sample was then dried under N_2_-stream. The residue was silylated using 150 µL Pyridine:BSTFA:TMCS (1:4:1) and analyzed with GC-MS similarly as lipophilic and hydrophilic extractives described above.

#### 3.5.3. Yield of Condensed Tannins

The phenolic concentration was expressed as milligrams of purified quebracho tannin equivalents to milligrams of dry extracts. It was assumed that the phenolic compounds in conifer barks measured by the Folin-Ciocalteu method primarily correspond to tannins, as reported by Bianchi et al. [24] and the expression “tannin yield” was used for the total phenolic compounds. Highly purified quebracho tannins (FINTAN QP, Silvateam S.p.A., San Michele Mondovì, Italy) was dissolved in 0.1 M NaOH. A series of different tannin concentration was prepared and a calibration curve was plotted against UV absorbance measured at 280 nm using a UV-Vis spectrophotometer (Shimadzu UV-2600). The extract powder was dissolved in 0.1 M NaOH and the absorbance at 280 nm was measured and the tannin content was calculated.

#### 3.5.4. Chemical Composition of Condensed Tannins

Condensed tannin, i.e., proanthocyanidins, was determined by HPLC after thiolytic degradation according to [69]. Briefly, freeze-dried samples were weighed (20–30 mg) into 1.5 mL Eppendorf vials and 1 mL of depolymerization reagent (3 g cysteamine/4 mL 13 M HCl / 56 mL methanol) was added. The vials were sealed and incubated for 60 min at 65 °C, after which the degradation products, i.e., free flavan-3-ols (terminal units) and their cysteaminyl derivatives (extension units), were separated on Zorbax Eclipse Plus C18 column (Agilent Technologies, Inc.; 2.1 × 50 mm, 1.8 μm) and determined by HPLC (Agilent 1290 Infinity, Agilent Technologies, Inc.) equipped with diode array detection (DAD) and fluorescence detection (FLD).

### 3.6. Lipid oxidation Inhibition Capacity of Tannin Extracts

The capacity of the plant extracts to inhibit lipid oxidation was assessed in a liposome model as described in [70] with some modifications. Briefly, soybean phosphatidylcholine liposomes were prepared according to [71]. Liposomes were stored at 4 °C at least one week prior to the study to increase the lipid hydroperoxide levels. The lipid oxidation reaction was carried out as described in [72,73]. Briefly, liposomes (100 µL) were mixed with sample, buffer (50 mM K-phosphate buffer pH 7.4, 100 mM glycine, and 450 μM ascorbic acid), and oxidative agent (150 µl of 1 mM adenosine diphosphate (ADP) in 25 μM FeCl_3_) at various sample concentrations. The suspension was allowed to react for 48 h at room temperature in the dark. Consequently, the concentration of the thiobarbituric acid reactive substances (TBARS) formed during the liposome oxidation was determined by a color reaction with thiobarbituric acid (TBA) and butylated hydroxytoluene (BHT). The color reaction was performed by mixing the oxidized liposome suspension with TCA/TBA solution (0.375% TBA, 2.25% TCA in 0.25 M HCl) and BHT (2% BHT in MeOH) and consequent incubation in a boiling water bath for 30 min. The solution was cooled to room temperature and centrifuged at 1710 × g for 10 min. Aliquots, 30 µL, of the supernatants were injected onto an Agilent 1100 HPLC-DAD with a SunFire C18 column (4.6 mm × 150 mm, 5 μm particle size, Waters co, Milford, MA, USA). Samples were eluted with a linear gradient (6–99% in 30 min) of acetonitrile in 0.05% trifluoroacetic acid, and the effluent was monitored at 532 nm. The concentration of malondialdehyde (MDA) was calculated against MDA-TBA standard curve (12.5–800 μM). Samples were analyzed in triplicate. Results from the liposome model are presented as inhibition efficiency ratio (IER) describing the inhibition percentage produced with sample concentration of 1 ng dm/mL.

### 3.7. Preparation of Meat Snack Samples

For the preliminary tests, a total of 5 000 g of fresh reindeer meat, free of visible fat and connective tissue, was chopped into small pieces. Each 1 000 g of the meat was mixed with 20 g of glucose, 20 g of vacuum salt, 0.4 g of starter culture (Bitec LS-25, Gewürzmüller^®^, FRUTAROM Savory Solutions GmbH, Korntal-Münchingen, Germany; including *Lactobacillus* and *Staphylococcus* species), and either (a) 1 g of dried extract, (b) 10 g of dried extract, (c) 10 g of liquid extract (corresponding to 0.95 g of dry matter in extract), or (d) 100 g of liquid extract (corresponding to 9.08 g of dry matter in extract). The mixture of ingredients was prepared into sausages, fermented, dried, and smoke-cured by using alder chips.

**Figure 6:**
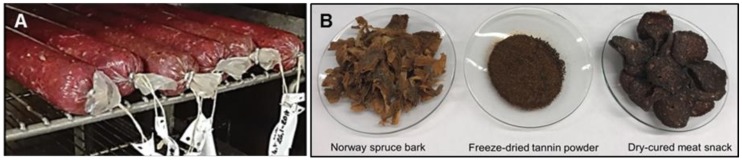
Preparation of fermented dry- and salt-cured reindeer meat snacks by using addition of tannin-rich extracts of Norway spruce bark.

For the final tests, the same basic recipe as described earlier was used with the total amount of 10 kg of reindeer meat. For each batch of 1 000 g of reindeer meat, the following tannin extracts were added: (a) a 5 g of dried tannin extract was mixed with the ingredients before fermentation, and (b) the dried tannin extract was diluted into an aqueous solution (with estimated concentration in the final product varying between ca. 0.2–0.4%) and sprayed after fermentation on dry-cured, sliced meat snacks (Figure 6).

### 3.8. Thiobarbituric Acid Test for Monitoring the Lipid Oxidation of Meat Snacks

The thiobarbituric acid (TBA) test was applied for monitoring the lipid oxidation in meat snacks according to the methodology by [74,75,76]. The TBA test was based on determination of fatty acid oxidation product malondialdehyde (MDA) from foodstuffs. In the TBA reaction, one MDA molecule reacts with the TBA molecule to form a pink color with an absorption maximum of 532–535 nm. The pH of the reaction solution was 2–3.

Ten g of freshly-prepared meat snack sample was weighed into 50 mL sample bottles. Butylated hydroxytoluene (BHT) solution was added (0.10% per expected fat content), followed by addition of 40 mL of cold (+4 °C) 10% trichloroacetic acid (TCA) solution, after which they were mixed with ultra-Turrax (13 000 r) / min) for 2 min, and the sample attached to the ultra-Turrax was rinsed back to the sample bottle by using 2 mL of 10% TCA solution. The sample mixture was then transferred to centrifuge tubes and the bottle washed with 5 mL distilled water, which is added to the centrifuge tubes. Centrifugation was done for 5 min at 3 000 rpm. The solution was then filtered through a Whatman no. 1 filter paper into a 50 mL graduated flask and filled with 10% TCA. 5 mL of filtrated TCA and 5 mL of TBA solution was then added into a volumetric flask that was mixed and incubated for 30 min in a 90 °C water bath. The sample flask was cooled for 10 min under running tap water. The samples were then poured into small glass beakers, from which taken into a syringe and pressed through a 0.2 µm filter into 10 mL tubes, and then measured with a spectrophotometer (Shimadzu 1800) using a quartz flow cuvette. The absorbance of the solutions was measured at 532 nm, using a blank solution of 5 mL of 10% TCA and 5 mL of TBA, also incubated at 90 °C. The standard curve was prepared similarly as the samples for concentrations of 0–30 mol/l, with an absorbance of 0.000–1000. The results from the standard curve were converted to mg TBA / kg of product.

### 3.9. Sensory Evaluation of Meat Snacks with Tannin Addition

The outlook, odor, taste, and structure of the randomized coded reindeer meat chips were evaluated by Luke’s laboratory panel (16 individuals, eight men, eight women) at Luke Jokioinen (Finland) sensory evaluation laboratory. The laboratory fills ISO 8589:2007 standard (ISO 8589:2007 Sensory analysis — General guidance for the design of test rooms). Scoring was done from 1–5 Evaluation points were classified as follows: 5 = reindeer chips were really good, 4 = good, 3 = not good/not bad, 2 = bad and 1 = really bad. The panelist was also asked about their willingness to buy the evaluated product (yes / no / maybe). The following four products were included in the randomized evaluation: (a) a meat snack with 0.5% tannin extract addition (i.e., extract 1); (b) the aforementioned meat snack for another time (i.e., extract 2); (c) a meat snack with sprayed extract (i.e., spray extract); and (d) the commercial control meat snack without tannin addition (i.e., control).

### 3.10. Statistical Analysis

The lipid oxidation inhibition capacity tests were performed as three replicates. The results are shown as arithmetic mean values ± standard deviations per extract type. The sensory evaluation results of the meat snack with and without tannin extract additions are shown as arithmetic mean values ± standard deviations per extract type. The results are presented as mean values based on all evaluators’ scores and as mean values for both genders (8 men and 8 women). The statistical analysis of inhibition capacity differences and differences in sensory evaluation results between the extracts were carried out by using a one-way ANOVA (IBM SPSS Statistics, v. 25; IBM Corporation, Armonk, NY, USA).

## 4. Conclusions

This study examined the capacity of conifer bark tannin extracts to prevent lipid oxidation by using a liposome model. According to our results, pine and spruce bark extracts exhibit high inhibition efficacies that were ten to hundred folds higher, respectively, than those of phenolic berry extracts. According to the sensory evaluation, the addition of tannin extracts did not significantly affect the smell and taste of the meat snacks. The findings indicate that conifer bark extracts may be used as sustainable food ingredients and/or special Nordic, “woody” aromas. However, more research is needed to allow their acceptance.

## Figures and Tables

**Table 1:** Composition and properties of proanthocyanidins in freeze-dried bark extracts.

Material	Concentration (g/100 g)	DP ^1^	PC ^2^ (%)	PD ^3^ (%)	A-type ^4^ (%)
Spruce WB	5.14 ± 0.10	4.8 ± 0.1	94.8	5.2	3.3
Spruce IB	6.75 ± 0.18	5.3 ± 0.4	94.7	5.3	3.5
Spruce OB	4.65 ± 0.02	4.4 ± 0.1	94.9	5.1	N.D.
Pine WB	14.04 ± 0.30	3.4 ± 0.1	100	N.D.	7.6
Pine IB	8.79 ± 0.17	3.5 ± 0.1	100	N.D.	8.4
Pine OB	8.87 ± 0.15	3.7 ± 0.1	100	N.D.	3.2
”Pettu”	10.01 ± 0.16	4.3 ± 0.1	100	N.D.	3.6

^1^ DP = mean degree of polymerization; ^2^ PC (%) = procyanidins, i.e., (epi)catechin units proportion; ^3^ PD (%) = prodelphinidins, i.e., (epi)gallocatechin units proportion; ^4^ A-type (%) = A-type bonding proportion from all bonding types (A-type + B-type); WB, whole bark; IB, inner bark; OB, outer bark; Pettu, commercial inner bark powder of pine.

**Table 2:** Results of sensory evaluation of reindeer meat snacks (mean ± SD in parenthesis).

Score ^a^					Would You Buy the Product?		
All evaluators, 16 individuals							
**Sample ^b^**	**Outlook**	**Smell**	**Taste**	**Structure**	**Yes**	**No**	**Maybe**
Extract 1	**3.48** (0.88)	3.58 (0.88)	3.56 (0.82)	3.33 (0.49)	5	11	
Extract 2	3.39 (0.74)	**3.60 (0.66)**	**3.65 (0.79)**	3.40 (0.77)	9	7	
Spray extract	3.38 (0.92	3.45 (0.91)	3.59 (0.76)	**3.45 (0.82)**	6	10	
Control	3.44 (0.72)	3.52 (0.73)	3.60 (0.91)	**3.45 (1.03)**	9	6	1
** Men, 8 individuals**							
**Sample**	**Outlook**	**Smell**	**Taste**	**Structure**	**Yes**	**No**	**Maybe**
Extract 1	3.58 (0.86)	3.06 (0.94)	3.69 (0.74)	3.10 (0.80)	4	4	
Extract 2	3.51 (0.86)	**3.07 (0.82)**	**3.80 (0.35)**	3.29 (0.46)	7	1	
Spray extract	3.60 (0.80)	2.92 (0.96)	3.77 (0.62)	**3.50 (0.92)**	5	3	
Control	**3.70** (0.76)	**3.07 (0.74)**	3.73 (0.58)	3.42 (1.06)	6	1	1
** Women, 8 individuals**							
**Sample**	**Outlook**	**Smell**	**Taste**	**Structure**	**Yes**	**No**	**Maybe**
Extract 1	**3.38 (0.93)**	4.10 (1.04)	3.42 (1.13)	3.56 (0.64)	1	7	
Extract 2	3.27 (0.65)	**4.12 (0.52)**	**3.49 (1.07)**	3.49 (1.04)	2	6	
Spray extract	3.15 (1.07)	3.98 (0.92)	3.40 (0.89)	3.40 (0.74)	1	7	
Control	3.17 (0.71)	3.97 (0.74)	3.47 (1.20)	3.47 (1.06)	3	5	

^a^ Scoring: 5, really good; 4, good; 3, not good/not bad; 2, bad; 1, really bad; ^b^ Extract 1 = 10 g of dry extract; Extract 2 = 10 g of liquid extract; Spray extract = liquid extract sprayed and dried on top of sliced meat snacks.

**Table 3:** Extraction conditions of Norway spruce and Scots pine bark condensed tannins, i.e., proanthocyanidins, by using pure hot water in a 2-litre stirrer reactor.

Material	Amount (g)	Temperature (°C)	Time (min)	Liquid/Solids (l/kg)	Stirring (rpm)
Spruce WB ^1^	2 × 200.0	90	120	7.13	200
Spruce WB ^2^	3 × 150.0	90	120	10	200
Spruce IB ^2^	150.0	90	120	10	200
Spruce OB ^2^	100.0	90	120	10	200
Pine WB ^2^	150.0	90	120	10	200
Pine IB ^2^	150.0	90	120	10	200
Pine OB ^2^	150.0	90	120	10	200

^1^ Kuru; ^2^ Ruotsinkylä; WB, whole bark; IB, inner bark; OB, outer bark.
